# Outbreak caused by the SARS-CoV-2 Omicron variant in Norway, November to December 2021

**DOI:** 10.2807/1560-7917.ES.2021.26.50.2101147

**Published:** 2021-12-16

**Authors:** Lin T. Brandal, Emily MacDonald, Lamprini Veneti, Tine Ravlo, Heidi Lange, Umaer Naseer, Siri Feruglio, Karoline Bragstad, Olav Hungnes, Liz E. Ødeskaug, Frode Hagen, Kristian E. Hanch-Hansen, Andreas Lind, Sara Viksmoen Watle, Arne M. Taxt, Mia Johansen, Line Vold, Preben Aavitsland, Karin Nygård, Elisabeth H. Madslien

**Affiliations:** 1 Norwegian Institute of Public Health, Oslo, Norway; 2 Municipality of Oslo, Norway; 3 Department of Microbiology, Oslo University Hospital, Oslo, Norway

**Keywords:** COVID-19, SARS-CoV-2, Omicron, outbreak investigation, cohort-study

## Abstract

In late November 2021, an outbreak of Omicron SARS-CoV-2 following a Christmas party with 117 attendees was detected in Oslo, Norway. We observed an attack rate of 74% and most cases developed symptoms. As at 13 December, none have been hospitalised. Most participants were 30–50 years old. Ninety-six percent of them were fully vaccinated. These findings corroborate reports that the Omicron variant may be more transmissible, and that vaccination may be less effective in preventing infection compared with Delta.

On 30 November 2021 the Norwegian Institute of Public Health (NIPH) was notified by a local laboratory in Oslo of a coronavirus disease (COVID-19) case with suspected severe acute respiratory syndrome coronavirus 2 (SARS-CoV-2) Omicron variant of concern (VOC) (Phylogenetic Assignment of Named Global Outbreak Lineages (Pangolin) designation B.1.1.529 BA.1) infection [1]. The laboratory provided information that the case was likely exposed at a company Christmas party held on 26 November 2021 and that one of the attendees had returned from South Africa on 24 November 2021. The novel SARS-CoV-2 variant Omicron was first detected in samples collected mid-November 2021 in Botswana and South Africa [2]. Here we describe the outbreak and preliminary findings from our epidemiological investigations.

## Setting

The closed event was held in a separate room (ca 145 m^2^) in a restaurant in Oslo from 18:00 to 22:30, after which the venue was opened to the public from 22:30 to 03:00. A pre-party had been arranged for the Christmas party attendees at a separate venue, after which they were transported by private buses to the restaurant. Although there were no restrictions in place for events at the time in Norway, all attendees of the party were reported to be fully vaccinated and had been asked by the organiser to perform a rapid antigen self-test. For other guests visiting the venue and employees working at the restaurant, there were no requirements for vaccination, COVID-19 testing, face-mask use or COVID certificate, and a guest list was not maintained. Attendees of the party mingled at the venue before and after dinner, following which the bar and dance area was opened to the public.

After detection of the outbreak on 30 November, all attendees at the party were requested by the municipality doctor in Oslo to self-quarantine at home for 10 days and to immediately take a PCR test. Those who tested positive were required to remain in isolation for at least 7 days [3]. In addition, a public message was released on 1 December asking anyone who had been at the venue from 22:30 on 26 November to 03:00 on 27 November to get tested by PCR as soon as possible, regardless of symptoms.

## Outbreak investigation

On 1 December, following a targeted exploration because of recent travel to South Africa, the suspected index case was verified to be infected with the SARS-CoV-2 Omicron VOC by whole genome sequencing (WGS) and the municipality of Oslo and the NIPH initiated an outbreak investigation. We conducted a cohort study to describe the outbreak and symptoms of infection in order to increase knowledge about the Omicron variant and to better target control measures.

The cohort was defined as all attendees at the party on 26 November 2021 (n = 117). A confirmed case was defined as a person who attended the party on 26 November 2021 and tested positive for the SARS-CoV-2 Omicron variant up to 13 December, through either PCR variant screening and/or WGS. A probable case was defined as a person who attended the party on 26 November and tested positive for SARS-CoV-2 by PCR. Contact details for the attendees were obtained from the organisers. Participants were interviewed by a team at the NIPH by telephone between 4 and 6 December, using a standardised questionnaire to collect demographic and clinical information, including date of onset and duration of a predefined list of symptoms, where relevant.

## Laboratory analysis

All SARS-CoV-2 positive samples from the party attendees were screened for SARS-CoV-2 virus variants in different local laboratories, mostly by S-gene 69/70 deletion or single-nucleotide polymorphism (SNP) targeted PCR for different other markers that were suited to differentiate Omicron from the hitherto completely predominant Delta variant. Available samples were also subjected to WGS. Whole genome sequences were submitted to Global Initiative on Sharing All Influenza Data (GISAID). Suspected outbreak index: EPI_ISL_7040235.

## Demographic and clinical information

In total, 111 out of 117 attendees (95%) participated in the interviews. Respondents had an average age of 39 years (SD: 9.2; median: 38; range: 26–68) and 48 (43%) of them were women. Most respondents (n = 107; 96%) were fully vaccinated. Eighty-nine percent of the respondents (n = 99) had received two doses of mRNA vaccines. None reported having received a booster dose. All respondents reported having a negative rapid antigen self-test taken at home or PCR within 1–2 days before attending the event. Eight (7%) respondents had previously had COVID-19, but none in the previous 4 months, according to information gathered through the interviews.

Of the 111 respondents, 66 (59%) were confirmed cases (26 based on WGS and 40 based on PCR VOC screening) and 15 (14%) were probable cases (PCR-positive only). One PCR-positive attendee was confirmed to be infected with SARS-CoV-2 Delta variant (Pango lineage B.1.617.2), and subsequently excluded from further analysis. The total attack rate for the Omicron variant was 74% (81/110) (Figure). The cases had an average age of 38 years (SD: 8.6; median 36, range: 26–61) and 35 (43%) were women. The remaining 29 attendees did not have a positive PCR result by 13 December 2021.

**Figure:**
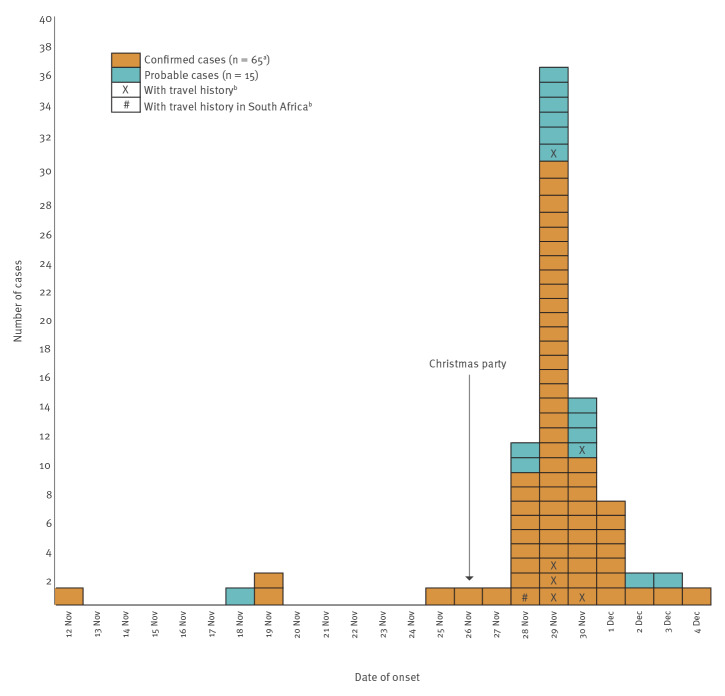
Distribution of COVID-19 cases infected with the SARS-CoV-2 Omicron variant by date of symptom onset and case classification, after attending Christmas party, Oslo, Norway, November−December 2021 (n = 81)

COVID-19: coronavirus disease; SARS-CoV-2: severe acute respiratory syndrome coronavirus 2.

^a^ One of the confirmed cases was asymptomatic and not presented in the epicurve.

^b^ Returned from travel to Africa or Europe less than 1 week before the party. None of them reported symptoms in the period before the party.

Assuming exposure occurred at the party, the incubation period for symptomatic cases ranged from 0 to 8 days with a median of 3 days (interquartile range: 3−4). One case was asymptomatic and 74 (91%) reported at least three symptoms. Among the 81 cases, the most common symptoms were cough (83%), followed by runny/stuffy nose (78%), fatigue/lethargy (74%), sore throat (72%), headache (68%) and fever (54%) (Table). When asked to grade the severity of symptoms on a scale from 1 (no symptoms) to 5 (significant symptoms), 42% (33/79) reported level 3 symptoms, whereas 11% (9/79) reported level 4 symptoms. None of the cases required hospitalisation up to 13 December 2021.

**Table:** Symptoms reported between 19 November and 3 December, by case status, in SARS-CoV-2 Omicron variant outbreak at Christmas party, Oslo, 26 November 2021 (n = 110)

Symptom	Non-cases (n = 29)		Cases (n = 81)^a^		
	n^b^	Median duration in days (IQR)^c^	n	%	Median duration in days (IQR)^c^
Cough	9	3 (2–6)	67	83	4 (3–5)
Runny/stuffy nose	10	4 (2–12)	63	78	4 (2–5)
Fatigue/lethargy	6	3 (2–4)	60	74	4 (2–5)
Sore throat	11	2.5 (1–6)	58	72	3 (2–5)
Headache	< 5	–	55	68	2 (2–4)
Muscle pain	< 5	–	47	58	2.5 (2–4)
Fever	< 5	–	44	54	2 (2–3)
Sneezing	10	4 (2–9)	35	43	3 (2–4)
Reduced smell	< 5	–	10	12	2 (2–3)
Reduced appetite	< 5	–	27	33	3 (2–5)
Heavy breathing	–	–	10	12	2 (1–5)
Reduced taste	< 5	–	19	23	2.5 (2–4)
Abdominal pain	< 5	–	5	6	2 (1–3)
Any of the above listed symptoms	19	4 (3–7)	80	99	6 (5–8)

IQR: interquartile range; SARS-CoV-2: severe acute respiratory syndrome coronavirus 2.

^a^ One confirmed case was asymptomatic and therefore not included.

^b^ Symptoms reported by less than five participants are reported as < 5.

^c^ Duration of symptoms might have been underestimated for some participants if the symptoms were still present after the day they were interviewed. Five non-cases and 62 cases reported still having symptoms on the day they were interviewed.

Duration of symptoms cannot be estimated accurately since 62 (78%) of the 80 symptomatic cases were still experiencing symptoms at the time of the interviews. Seven cases reported symptoms consistent with COVID-19 within 2 weeks prior the party, with less than five reporting onset more than 1 week before the party.

Seven cases reported recent (≤ 7days) travel abroad, three in Africa and four in Europe. None of these cases reported symptoms in the period before the party. Almost all (n = 104; 95%) of the 110 respondents reported being at work the week before the party and 87% (n = 96) shared offices.

## Vaccination status

Most of the cases (n = 79; 98%) and non-cases (n = 27; 93%) were fully vaccinated with a median time since receiving the last vaccine dose of 79 days for cases and 87 days for non-cases (no statistically significant difference, Wilcoxon rank-sum p = 0.48). Among cases who had received two vaccine doses, 55% (41/75) received Comirnaty (BNT162b2 mRNA, BioNTech-Pfizer, Mainz, Germany/New York, United States (US)) whereas 23% (17/75) received Spikewax (mRNA-1273, Moderna, Cambridge, US). Among the 25 non-cases who had received two doses, seven received Comirnaty whereas 12 received Spikewax.

In addition, during the investigation and as at 13 December, we detected nearly 70 other guests that were likely infected at the venue, and the Omicron variant was detected in 53 of these through PCR variant screening or sequencing.

## Discussion

Our preliminary investigation of the first outbreak in Norway with the Omicron VOC supports the notion that this SARS-CoV-2 variant is highly transmissible even among fully vaccinated people. Given the limited knowledge on transmissibility, severity and immune escape for this VOC [2,4], it will be important to compare outbreaks in different contexts. Superspreading events have been widely reported linked to other variants [2,5-8]. Previous outbreaks caused by the SARS-CoV-2 Delta variant have also been reported in Norway among highly vaccinated populations (data not shown). In the outbreak described here, the high attack rate with symptomatic infection was likely exacerbated by the context and setting of the outbreak (indoor location, long exposure time, crowding, and the need to talk loudly) [6,9]. Some transmission may have occurred among colleagues in the workplace or at the pre-party as some of the attendees had symptoms prior to the party. We can also not exclude that there may have been multiple virus introductions, although all attendees reported a negative test result before the party. This will further be explored when whole genome sequencing results are available for all confirmed cases. However, our experience with this outbreak corroborates reports from other countries that the Omicron variant may be more transmissible and that vaccination may be less effective to prevent infection than for the Delta variant [4,10-13]. It is not possible to draw any conclusions regarding effectiveness of different vaccine types against infection with Omicron based on this single outbreak.

Assuming attendees were infected at the party, we observed a median incubation period of 3 days, which is short compared with previous reports for Delta and other previously circulating non-Delta SARS-CoV-2 (4.3 and 5.0 days, respectively) [14]. Furthermore, almost all cases developed at least one symptom, and more than half (54%) reported fever. Attendees will be interviewed at least once more in the coming days in order to follow up on symptoms, duration and secondary transmission to household members.

The outbreak was rapidly detected due to the comprehensive and timely test capacity and surveillance in Norway. The combination of rapid detection with contact tracing, isolation and quarantine measures, was probably able to slow down the spread of the outbreak in the short term, but it is unlikely that the implemented control measures will have interrupted the spread of the Omicron VOC. Norway does not currently apply the European Union Digital COVID Certificate for entry to venues. We believe that this outbreak would probably not have been prevented even if such a system were in place, given that nearly all participants had received at least one dose of the COVID-19 vaccine and the majority were fully vaccinated and tested by antigen test before the event. However, we cannot exclude that vaccination has reduced the risk of serious illness; no hospitalisations were reported among cases until 13 December, and until more information is available, vaccination, including providing booster doses to risk groups, will continue to be a key control measure. In addition, it is important to further strengthen communication about the need to stay at home when experiencing symptoms, regardless of the cause.

In light of the rapidly evolving situation, Norway has implemented increasingly stringent control measures since 2 December, because of both stress on the healthcare system due to the Delta variant and the rapid spread of the Omicron VOC. As at 16 December, these measures include restrictions of public and private events, such as reduction of group sizes, requirements for working from home and a ban on serving alcohol in restaurants and bars for an initial period of 4 weeks [15]. 

## Conclusions

The preliminary results of our outbreak investigation indicate that the SARS-CoV-2 Omicron VOC is highly transmissible among fully vaccinated young and middle-aged adults. However, given the specific context of the outbreak in a high-risk setting for transmission, the findings must be interpreted with caution. The investigation is continuing to determine the full spectrum of illness and its duration, the risk factors for infection and the extent of secondary transmission. More systematic surveillance and research is needed in order to determine the epidemiological and clinical characteristics of the Omicron variant and the appropriate control measures for managing outbreaks.
